# A novel recombinant baculovirus overexpressing a *Bacillus thuringiensis* Cry1Ab toxin enhances insecticidal activity

**DOI:** 10.1186/1480-9222-16-7

**Published:** 2014-04-15

**Authors:** Wael El-Menofy, Gamal Osman, Abdulrahman Assaeedi, Mohamed Salama

**Affiliations:** 1 Agricultural Genetic Engineering Research Institute (AGERI) - ARC, 9 Gamaa St, Giza, Egypt; 2 Biology Department, Faculty of Applied Sciences, Umm Al Qura University, Makka 21955, PO Box 715, Kingdom of Saudi Arabia; 3 Faculty of Science, Ain Shams University, Cairo, Egypt

**Keywords:** *Bacillus thuringiensis*, Toxin, Baculovirus, Cry 1Ab, LC_50_

## Abstract

Baculoviruses have been genetically modified to express foreign genes under powerful promoters in order to accelerate their speed of killing. In this study a truncated form of cry1Ab gene derived from *Bacillus thuringinsis* (Bt) subsp. *aegypti* isolate Bt7 was engineered into the genome of the baculovirus *Autographa californica* multiple nuclearpolyhedrosis wild type virus, in place of the polyhedrin gene by using homologous recombination in *Spodoptera frugiperda* (Sf) cells between a transfer vector carrying the Bt gene and the wild type virus linearized DNA. Recombinant wild type virus containing the cry1Ab gene was detected as blue occlusion-negative plaques in monolayers of Sf cells grown in the presence of X-Gal. In Sf cells infected with plaque-purified recombinant virus, the cry1Ab gene was expressed to yield a protein of approximately 82-kDa, as determined by immunoblot analysis. The toxicity of the recombinant virus expressing the insecticidal crystal protein (ICP) was compared to that of the wild-type virus. Infected-cell extract was toxic to cotton leaf worm *Spodoptera littoralis* second instar larvae and the estimated LC_50_ was 1.7 μg/ml for the recombinant virus compared with that of wild-type virus which was 10 μg/ml.

## Introduction

The development of synthetic pesticides since 1940 coupled with the improvement in chemical applications technology dramatically increased the potential for agricultural pest control [1]. It did not take long before people began to see the shortcomings of this new technology. Pests that had been naturally controlled by predators and parasites began to cause significant damage and became resistant to chemical pesticides [2]. Moreover, chemical pesticides have become expensive and imposed greater hazard to the environment and all living organisms [3]. An alternative strategy for effective control of pests is the use of biological insecticides either by itself or within an integrated pest management program (IPM) [4,5]. The benefit of biological control by viruses, especially baculoviruses has become apparent in recent years. Baculoviruses are major insect pathogens and are characterized by the presence of a large circular double stranded DNA genome and enveloped rod shaped virions. Baculoviruses and their use as biological insecticides have been studied for several years [6]. Since baculoviruses are host-specific and do not infect vertebrates or plant species, and considered to be the best among viruses for insect pest control [7]. More than 600 insect species serve as hosts for baculoviruses, which belong to family baculoviridae [8]. They are insect pathogens and cause fatal disease in insects mainly in members of Lepidopteran order [9-11]. However, wild type baculoviruses have several limitations in their use as biological control agents [12]. The major problems are the slow speed of action, low virulence to older instar larvae and sensitivity to UV-light [13]. Development of genetically engineered viruses has resulted in an enhancement of the speed with which baculoviruses kill target pests by introducing additional pesticidal genes into the genome of *Autographa californica* nucleopolyhedrovirus wild type virus. These recombinant baculoviruses were evaluated under laboratory conditions for their improved pesticidal properties [7,14]. Because the C-terminal half of 135-kDa Cry1 is not toxic, it could be eliminated and use only the N-terminal half, in the same time the truncated proteins do not form inclusions. These insecticidal transgenes included insect hormone genes which disturb the physiological hormonal balance of the insect [15,16] and Bt gene(s). [1,17,18]. Moreover, the production of recombinant baculoviruses expressing specific neurotoxin proteins such as mite toxin and the scorpion toxin [19-21] enhanced the biological activity of baculoviruses. This study aims to introduce a truncated cry1Ab gene from *Bacillus thuringiensis* into a baculovirus in order to enhance its insecticidal activity.

## Materials and methods

### Bacterial strains

*Bacillus thuringiensis* local Egyptian isolate Bt7 was provided by Prof. G. Osman Agricultural Genetic Engineering Research Institute (AGERI). *Escherchia coli* strain XL-1 blue (Stratagene Inc. 1 north state st suite 900, Chicago, 606062, USA) was used as the host for plasmid DNA preparation and propagation.

## Virus

*Autographa californica* nucleopolyhedrovirus wild type virus, strain E2 [22] was used as a wild type (wt) virus (supplied by Invitrogen, 3175 Staley Road, Grand Island, NY 14072, USA).

### Insects cell culture

*Spodoptera frugiperda* cell line IPLB SF21-AE (Sf 21) was kindly provided by Dr. Hussen, A. Virology Dept. Faculty of Veterinary, Cairo University, Giza, Egypt. Sf21 cells were maintained at 27°C and grown in a TNM-FH medium that was supplemented with 10% (w/v) fetal bovine serum and antibiotics, and subcultured every 3 to 4 days.

### Insects

Cotton leaf worm (*Spodoptera littoralis* – Boisd – Noctuidae - Lepidoptera), was provided by Insectary of (AGERI), Giza, Egypt. The Egyptian cotton leaf worm *Spodoptera littoralis* (Biosd) were reared in insectary of AGERI under highly controlled conditions the larvae were fed on semisynthetic diet described by Shorey and Hale [23], the insect culture were maintained on 25 + or - 2°C, 65 - 70% RH and natural photoperiod.

### Construction of a transfer vector containing the truncated cry1Ab gene

Cry1Ab toxin was chosen because of its high insecticidal activity against Spodoptera sp. The truncated form of the cry1Ab gene, encoding the 82 kDa toxin protein was cloned from Bt7 strain followed by determination of its DNA sequences. The truncated cry1Ab gene was amplified using one pair of specific primers (WG1 and WG2) based on the nucleotide sequence of the published cry1Ab gene.The cry1Ab fragment 2.2 kb was amplified by using a pair of specific primers i.e.WG1 forward (5′ATGGATAACAATCCGAACATC3′) and WG2 reverse (5′ TAGCGTAACGTAATT CTCTTT 3′). The primer was designed according to cry1Ab gene in gene bank accession number: gbKF93868.2.1. The amplified DNA fragment was cloned into pGEM-T easy vector (Promega, 2800 Woods Hollow Road, Madison, WI 53711 USA ). The new construct was named wpGM-Bt7. The wpGM-Bt7 was subcloned into pBlueBacIII transfer vector (Invitrogen) by restriction digestion using *Nco*I and *Pst*I endonucleases. The newly constructed plasmid named wpBac-Bt7.

### Co-transfection of *Spodoptera frugiperda* cells and isolation of recombinant virus

Recombinant wild type baculovirus, containing the cry1Ab gene under transcriptional control of the polyhedrin promoter was introduced by *in vitro* homologues recombination as described in the Invitrogen instruction manual. Linear viral DNA (500 ng) was co-transfected with (1-2 ug) wpBac-Bt7 containing the cry1Ab into *S. frugiperda* Sf21 cells using liposome-mediated transfection method according to Webb and Summers [24]. After 48 h the medium from transfected cells was collected. For plaque assay screening, three viral serial dilutions (10^-1^, 10^-2^ and 10^-3^) were mixed with 1 × 10^6^ *S. frugiperda* cells Sf 21 and seeded in complete TC-100 medium in 60 mm dishes (Falcon Invitrogen) for 1 h. After incubation, the media were completely removed from all plates and the infected cells were overlaid with the supplemented TC-100 medium containing 0.5% baculovirus agarose and 150 μg ml^-1^ X-gal (5-bromo-4-chloro-3-indolyl-ß-D-galactopyranoside) as a chromogenic substrate. The plates were incubated at 27°C for five days and screened for the presence or absence of polyhedra by light microscopy. Plaque forming units (pfu) were detected as blue spots after five to six days post-infection (p.i.) and polyhedrin negative blue plaque were visualized using an inverted light microscope. The presence of Bt gene cry1Ab in the recombinant virus AcW-Bt7 were confirmed by PCR using WG1 and WG2 specific primers.

### Analysis of protein expression by SDS-PAGE and Immunoblotting

Insect cells were infected with AcW-Bt7 at a multiplicity of infection (moi) of (10 moi/cell) recombinant virus. The expression of the crystalline protein of cr1Ab in the insect cells was detected by SDS-PAGE and immunoblotting assay. Sf cells infected with the recombinant virus were lysed in electrophoresis sample buffer (0.06 mol ^-1^ Tris-HCl pH 6.8, 2% SDS, 10% glycerol, 5% b-mercaptoethanol and bromophenol blue). Samples were denatured for five min in boiling water and separated in 10% polyacrylamide gel [25]. Gels were stained with 0.1% Coomassie blue R-250 in 7% (v/v) acetic acid and 50% methanol. The gels were destained in 7% (v/v) acetic acid with 50% methanol. For immunodetection, proteins were transferred using a semi-dry blotter (Bio-Rad) on to a PVDF membrane (Millipore) and probed with an antibody directed against the Cry1A protein. The immune-detection was performed using the ECL kit from Amersham Bioscience, church farm business park, corston bath Ba2 9Ap, UK.

### Bioassay of the recombinant virus

The bioassay of infected Sf 21 cells with recombinant virus against second instar of cotton leaf worm compared to the wild type virus. Sf 21 cells were infected at an moiof 10 and collected at 72-h post infection (p.i.). For each virus, the cells were washed twice with PBS freeze-thawed several times to disrupt the cell membrane then the samples were diluted over a concentration range of 1.5-300 μg ml^-1^ of polyhedra then assayed against second instar larvae of cotton leaf worm (CLW) as described by Ibara and Federici [26]. For each viral concentration Twenty second instar larvae were added in each cup. Bioassays were repeated three times. Mortality was scored daily until death or pupation. The LC_50_ was determined by probit analysis plot [27]. Control treatments consisted of uninfected larvae and infected larvae with wild type virus.

## Results

### Cloning of *Bacillus thuringiensis* cry1Ab Gene

PCR was carried out to amplify a 2.2 kb fragment by using whole Bt7 genomic DNA as a template. The PCR-amplified DNA fragment was purified and cloned in TA cloning vector (pGEM-T-Easy vector, Promega). Screening for the positive clones (white colonies) was done using of both single and double restriction enzyme digestion. The new construct was named wpGM-Bt7. DNA sequencing of cry1Ab gene was carried out using the automated sequencer ABI PRISM 310. The sequence analysis of cry1Ab indicated that the amplified fragment is a typical Bt gene with low G + C content (37.63%) and high A + T content (62.37%). Moreover, the nucleotide sequence alignment of the cry1Ab gene revealed that the cry1Ab of Bt7 had an identity of 98% with the Genbank cry1Ab sequence while it was 100% identity with the Genbank at amino acid level. The sequence of cry1Ab gene of the isolate was submitted to GenBank with the accession #KC581790.

### Sub-cloning of cry1Ab into pBlueBacIII baculovirus transfer vector

cry1Ab was subcloned into non-fused pBlueBacIII baculovirus transfer vector under the control of the polyhedrin gene promoter. Screening of the baculovirus hybrid plasmids using double digestion with *Nco*I and *Pst*I endonucleases showed an 2.2 kb DNA fragment corresponding to the expected size of the cry1Ab gene. The resulting hybrid plasmid was named wpBac-Bt7.

### Generation and purification of genetically modified wild type virus containing cry1Ab gene

**Figure 1:**
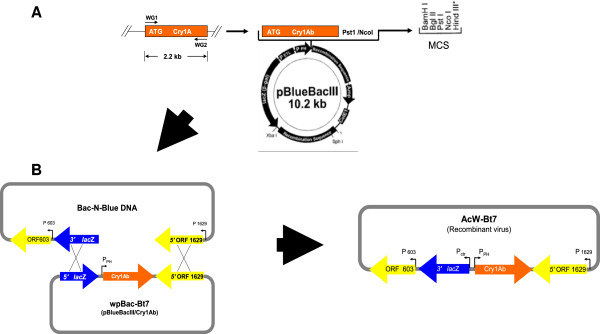
**Schematic diagram of the recombinant virus AcW-Bt7 construction. (A)** The Cry1Ab gene (2.2 kb) was PCR amplified using WG1 and WG2 specific primers introducing PstI and NcoI restriction sites to its 5*'* and 3*'* ends, respectively. The digested Cry1Ab-PstI/NcoI fragment was cloned into Pst/NcoI sites of pBlueBacIII transfer vector generating the recombinant plasmid wpBac-Bt7. The features of pBlueBacIII carrying the truncated form of Cry1Ab gene under control of Polyhedrin promoter are shown. **(B)** Upon co-transfection of the wpBac-Bt7 transfer vector and the linearized Bac-N-Blue DNA into Sf9 cells, homologous recombination between ORF1629 sequences and lacZ sequences are occurred generating the recombinant virus AcW-Bt7.

**Figure 2:**
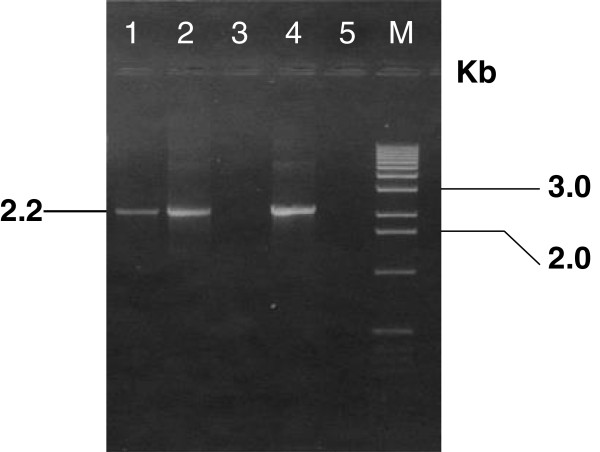
**Ethidium bromide-stained 1% agarose gel showing the amplified *cry1Ab *fragment using: Lane 1: total DNA of strain Bt7.** Lane 2: wpBac-Bt7 recombinant plasmid (positive control). Lane 3: wt *Ac*NPV DNA (negative control). Lane 4: *Ac*wBt7 DNA recombinant virus. Lane 5: PCR negative control without template. M: 1 kb plus ladder DNA marker.

**Figure 3:**
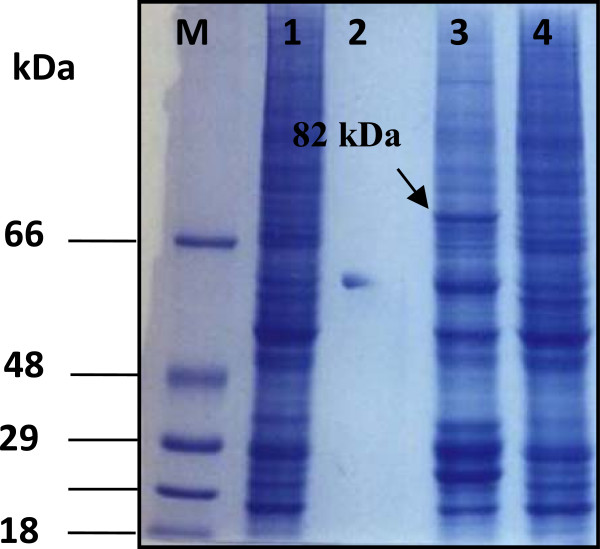
**SDS-PAGE of total protein profile of I × 10**^**6 **^***S. frugiperda *infected cells with recombinant virus shows: Low range protein marker (M).** Lane 1: mock-infection. Lane 2: Cry1Ab protein as a positive control. Lane 3: Sf cells infected with recombinant virus AcWBt7. Lane 4: Sf cells infected with wild type virus.

**Figure 4:**
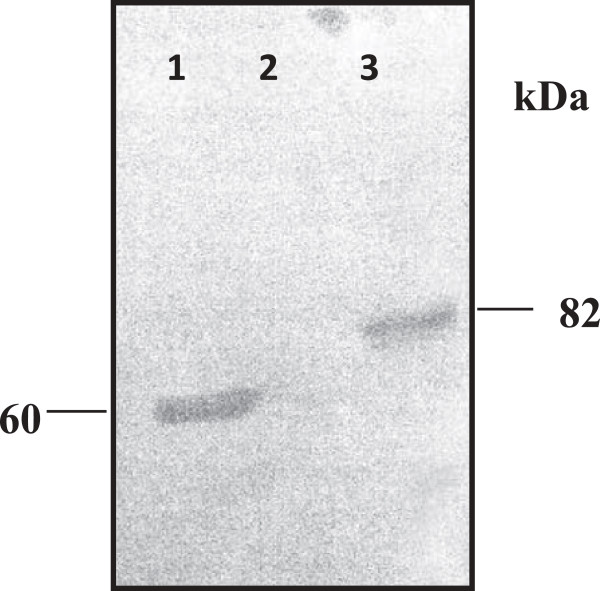
**Western blotting of approximately 5 × 10**^**4 **^***S. frugiperda *21 cells showing the detection of Bt Cry1Ab toxin 82-kDa after 48 hrs p.i.** Lane 1: purified Cry1Ab toxin (positive control), lane 2: cells infected with *wt Ac*NPV (negative control) and lane 3: cells infected with *Ac*wBt7 recombinant virus.

Wild type baculovirus was chosen to be the parental virus for generation of recombinant virus by using the homologous recombination method. Sf cells were co-transfected with baculovirus hybrid plasmid wpBac-Bt7 and the linearized wild type virus DNA to facilitate the replacement of the cry1Ab gene (located in the hybrid plasmid) in place of the polyhedrin gene (located in the wild type baculovirus genome) via homologues recombination in the presence of Cellfectin cationic liposome reagent (Figure 1). Recombinant virus was distinguished from the wild type virus by using the plaque assay method [28]. Four blue plaques were selected. They indicated the occurrence of homologues recombination within the insect cell and resulted in a recombinant virus which carried the Bt cry1Ab gene with blue plaque phenotype and named AcW-Bt7. This result was further confirmed by detection of cry1Ab gene through PCR (Figure 2). SDS-PAGE of the total protein extract of infected cells (Figure 3) and also Western blot analysis of the protein profile by using a polyclonal antibody specific for the Cry1Ab protein, confirmed the presence of the 82 kDa protein band, which reacted successfully with the Cry1A specific antibody (Figure 4).

### Insect toxicity and bioassay of the recombinant baculovirus

**Figure 5:**
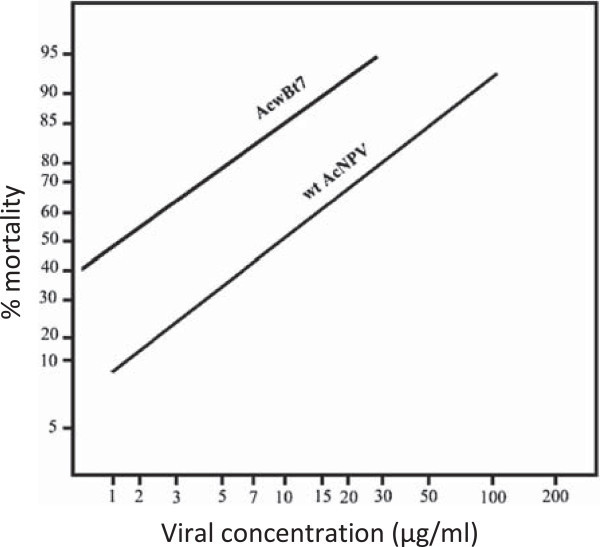
**LC**_**50 **_**regression line of *S. littoralis *** **2**^**nd **^**instar larval response to different viral concentrations of the recombinant virus (*Ac*** **wBt7) and the wild type virus.**

The biological activity of the AcwBt7 recombinant virus expressing Bt Cry1Ab toxin protein was evaluated against cotton leaf worm *Spodoptera littoralis* 2^nd^ instar larvae. The LC_50_ value for Acw-Bt7 and the wild type virus were determined by feeding of 2^nd^ instar larvae of cotton leaf worm on a semi-artificial diet with different concentrations of the final whole culture (FWC) ranging from 1.5-300 μg ml^-1^ for both the recombinant virus Acw-Bt7 and virions of the wild type virus. The results of the bioassay revealed that the LC_50_ value for the recombinant virus was 1.7 μg ml^-1^, compared to the LC_50_ value for the wild type virus which was 10 μg ml^-1^. Regression analysis of the cotton leaf worm larval response to Acw-Bt7 (recombinant virus) and wild type virus are illustrated in (Figure 5). The LC_50_ was determined by probit analysis plot [27]. Control treatments consisted of uninfected larvae and infected larvae with wild type virus.

These result of the bioassay revealed that the recombinant virus is five-fold more effective than the wild type.

## Discussion

Because the C-terminal half of 135-kDa Cry1 is not toxic, it could be eliminated and use only the N-terminal half, in the same time the truncated proteins do not form inclusions. A recombinant virus containing the truncated cryIAb gene from *Bacillus thuringiensis* Bt7 was constructed successfully. The insecticidal crystal protein (ICP) Cry1Ab of 82 KDa was produced in the infected Sf cells. These results proved that wild type baculovirus can be used to express and study the properties of the insecticidal *Bacillus thuringiensis* protein yielding information relevant to an understanding of the molecular biology of this protein as well as to improve the baculovirus insecticidal activity. These results agree with the findings of [29,30], who introduced a truncated (*orf*) gene coding for the N-terminal 645 amino acids of the protoxin in a similar way into a wild type virus to avoid crystal formation. This protein produced in considerable amounts was biologically active, but as expected, did not precipitate into crystals confirming that the C-terminal part of the Cry1Ab ICP is required for crystal formation [31,32]. Chang et al. [30] noted also that the complete cry1Ab open reading frame (*orf*) crystal protein expressed by *Ac*NPV/JM3 recombinant virus from the polyhedrin promoter in *Sf* cells was highly toxic to *P. brassicae* larvae. These results indicated that the wild type baculovirus-expressed ICP is authentic and could in principle enhance the insecticidal action of a recombinant baculovirus. The results of the bioassay revealed that the LC_50_ value for the recombinant virus was 1.7 μg ml^-1^, compared to the LC_50_ value for the wild type which was 10 μg ml^-1^. These results of the bioassay revealed that the recombinant virus is 5-fold more effective than the wild type virus, and this results will encourage us to test this recombint virus on other lepidoperan pests. This is in agreement with the results of [33-35], who noted that the level of expression of the Cry1Ab and Cry1Ac proteins in insect cells infected with the occluded viruses (p10- based promoter) *Ac*OBtm and *Ac*OBt73 had at least 1/5 of the expression of the same proteins in insects infected with the non-occluded viruses (polyhedrin-based promoter), *Ac*Btm and *Ac*Bt73. This lower expression could be due to the simultaneous expression of the polyhedrin gene, since they might compete for the same resources or transcription factors inside the cell. The toxic action of Bt *in vivo* is known to be dependent on the binding of activated toxin to the external surface of midgut microvilli, where the toxins appear to bind to specific proteins receptors [36,37] and then intercalate forming transmembrane cation pores [38] that lead to cell death. However, it has been shown that the Cry1A and CryIIIA toxins can insert into planar lipid bilayers that have no protein receptors [30,39]. Moreover, evidence from patch clamp studies of the action of the Cry1C toxin on *Sf* cells indicated that this Bt toxin may act inside the cell and is capable of inserting into the cell membrane from the cytoplasmic side [40,41]. These results are consistent with that of [8,42]), who reported that the normal targets of the δ-endotoxin are the gut epithelial cells. However, several other types of cell have also been shown to have receptors for the toxins [11,43]. Characterization of factors determining the host-range of the baculoviruses may lead to greater potential for manipulation of the host-range. Considering that the use of baculoviruses may increase substantially in the next 10 years [44]. With growing awareness of environmental issues associated with expanding the demand for effective bioagents although Baculoviruses are essentially nonpathogenic to mammals as well Bt these may be need further study in terms of Biosafety of recombinant baculovirus as a result of viral genome alteration.

## Competing interest

The authors declare that they have no competing interest.

## Authors’ contributions

Developing original approach (GO), improving and confirming the technique (MS, GO), performing experiment (GO, WE, MS), analyzing data (WE, GO, AA), writing the manuscript (AA, GO), molecular genetic studies (GO, WE), participated in the sequence alignment and drafted the manuscript (GO, AA). All authors read and approved the final manuscript.
